# Nitric oxide-mediated inhibition of phenylephrine-induced contraction in response to hypothermia is partially modulated by endothelial Rho-kinase

**DOI:** 10.7150/ijms.39074

**Published:** 2020-01-01

**Authors:** Soo Hee Lee, Seong-Ho Ok, Raghavendra Baregundi Subbarao, Ji-Yoon Kim, Sung Il Bae, Yeran Hwang, Seongyeong Tak, Ju-Tae Sohn

**Affiliations:** 1 Department of Anesthesiology and Pain Medicine, Gyeongsang National University College of Medicine, Gyeongsang National University Hospital, 15 Jinju-daero 816 beon-gil, Jinju-si, Gyeongsangnam-do, 52727, Republic of Korea.; 2 Department of Anesthesiology and Pain Medicine, Gyeongsang National University Changwon Hospital, Changwon, 51427, Republic of Korea.; 3 Department of Anesthesiology and Pain Medicine, Gyeongsang National University Hospital, 15 Jinju-daero 816 beon-gil, Jinju-si, Gyeongsangnam-do, 52727, Republic of Korea.; 4 Institute of Health Sciences, Gyeongsang National University, Jinju-si, 52727, Republic of Korea.

**Keywords:** hypothermia, nitric oxide, phenylephrine, endothelial Rho-kinase, contraction, aorta, phosphoinositide 3-kinase

## Abstract

This study examined the possible upstream cellular signaling pathway associated with nitric oxide (NO)-mediated inhibition of phenylephrine-induced contraction in isolated rat aortae in response to mild hypothermia, with a particular focus on endothelial Rho-kinase. We examined the effects of mild hypothermia (33°C), wortmannin, N^ω^-nitro-L-arginine methyl ester (L-NAME), Y-27632, 1H-[1,2,4]oxadiazolo[4,3-a]quinoxalin-1-one (ODQ) and methylene blue, alone and combined, on phenylephrine-induced contraction in isolated rat aortae. Finally, we examined the effects of mild hypothermia, wortmannin, Y-27632 and L-NAME, alone and combined, on endothelial nitric oxide synthase (eNOS) and endothelial Rho-kinase membrane translocation induced by phenylephrine. Mild hypothermia attenuated phenylephrine-induced contraction only in endothelium-intact aortae. L-NAME, wortmannin, ODQ and methylene blue increased phenylephrine-induced contraction of endothelium-intact aortae pretreated at 33°C. Wortmannin did not significantly alter the L-NAME-induced enhancement of phenylephrine-induced maximal contraction of endothelium-intact aortae pretreated at 33°C. Wortmannin abolished the ability of Y-27632 to magnify the hypothermic inhibition of maximal phenylephrine-induced contraction. Wortmannin and L-NAME inhibited the enhancing effect of mild hypothermia on phenylephrine-induced eNOS phosphorylation. Y-27632 and L-NAME attenuated the enhancing effect of hypothermia on phenylephrine-induced endothelial Rho-kinase membrane translocation. The results suggest that hypothermia-induced, NO-dependent inhibition of phenylephrine-induced contraction is mediated by phosphoinositide 3-kinase and inhibited by endothelial Rho-kinase activation.

## Introduction

Hypothermia may exert a neuroprotective effect in traumatic brain injury 1,2. The alpha-adrenoceptor agonist phenylephrine can be used to improve cerebral perfusion pressure that has been compromised by hypothermia-induced hypotension, which is regarded as a side effect of hypothermia 1,2. Mild hypothermia (34°C) attenuates phenylephrine-induced contraction by increasing nitric oxide (NO) production 3. In addition, norepinephrine-induced contraction is attenuated by hypothermia in isolated rat aortae, and this attenuation is inhibited by removal of the endothelium 4. Moderate hypothermia (25 to 31°C) reduces phenylephrine-induced contraction via the NO-cyclic guanosine monophosphate (cGMP) pathway 5. Endothelial phosphoinositide 3-kinase (PI3K)-induced NO production modulates phenylephrine-induced contraction in endothelium-intact aortae, and this effect seems to be inhibited by endothelial Rho-kinase 6,7. In addition, endothelium-dependent NO-induced relaxation is mediated partially by inhibition of the RhoA and Rho-kinase pathways in endothelium-intact arteries 8. The Rho-kinase inhibitor Y-27632 inhibits phenylephrine-induced contraction only in endothelium-intact rat aortae, whereas pretreatment with NO synthase inhibitor N^ω^-nitro-L-arginine methyl ester (L-NAME) attenuates Y-27632-mediated inhibition of phenylephrine-induced contraction 9. Furthermore, NO-mediated vasodilation of cavernosal arterioles can overcome Rho-kinase-mediated vasoconstriction to produce penile erection 10. The cholesterol-lowering drugs known as statins inhibit 3-hydroxy-3-methylglutaryl-coenzyme A, thereby inhibiting the synthesis of isoprenoids (geranyl-geranyl pyrophosphate) and cholesterol, leading to inhibition of Rho-kinase and increased NO production 11. Combined treatment with the Rho-kinase inhibitor fasudil and statins prevents cerebral spasm after subarachnoid hemorrhage 12. However, exposure to cold temperatures causes vessels to constrict via a mechanism involving reactive oxygen species-induced activation of Rho-kinase, alpha-2 adrenoceptor translocation and decreased NO production 13. Cold-induced activation of Rho-kinase also causes vasoconstriction via the inhibition of myosin light chain phosphatase (MLCP) and decreased NO production 13. Taken together, these previous reports suggest that hypothermia-induced Rho-kinase activation inhibits NO-dependent vasodilation 8-13. Therefore, we tested the hypothesis that endothelial Rho-kinase inhibits hypothermia-induced NO-mediated attenuation of phenylephrine-induced contraction 6-13. The goal of this study was to examine the possible upstream cellular signaling pathway associated with hypothermia-induced NO-mediated attenuation of phenylephrine-induced contraction in the isolated rat aorta, with a particular focus on endothelial Rho-kinase.

## Materials and Methods

The experimental protocol and methods were approved by the Institutional Animal Care and Use Committee of Gyeongsang National University. All the experimental protocols complied with the regulations in the Guideline for the Care and Use of Laboratory Animal prepared by Gyeongsang National University.

### Preparation of rat thoracic aortae and subsequent isometric tension measurement

Isolated rat thoracic aortae were prepared for isometric tension measurement as described previously by our laboratory 14,15. Male Sprague-Dawley rats (250-300 grams) were euthanized with 100% carbon dioxide supplied via a small hole in the cage. After the descending thoracic aorta of each rat was removed from the thorax, the perivascular connective tissue and fat were removed under a microscope. As there are regional differences in vasoreactivity to acetylcholine in endothelium-intact rat aortae, the descending thoracic aorta was used throughout the current study 16. Each aorta was cut into small aortic rings measuring 2.5 to 3 mm in length. These rings were suspended in a Grass isometric force transducer (FT-03, Grass Instrument Co., Quincy, MA, USA) with a resting tension of 3.0 in a 10-mL organ bath full of 37°C Krebs solution. The resting tension of 3.0 g was maintained for 120 min to reach equilibrium. The Krebs solution was exchanged for fresh Krebs solution every 30 min. The endothelium of some isolated rat thoracic aortae was removed to produce endothelium-denuded rat thoracic aortae. To remove the endothelium from isolated rat thoracic aortae, two 25-gauge needles were inserted into the lumen of the aorta, and the aorta was then rotated around the two needles. To confirm endothelial denudation, we induced contraction by adding phenylephrine (10^-8^ M) to the organ bath. After phenylephrine produced a sustained and stable contraction, acetylcholine (10^-5^ M) was added to the organ bath to assess endothelium-dependent relaxation, and less than 15% acetylcholine-induced relaxation was considered to indicate endothelium denudement. To confirm endothelial integrity, we added phenylephrine (10^-7^ M) to the organ bath. After phenylephrine (10^-7^ M) produced a stable and sustained contraction in the endothelium-intact rat aorta, acetylcholine (10^-5^ M) was added to the organ bath to assess endothelial integrity. In this experiment, more than 80% acetylcholine-induced relaxation was considered to indicate that the endothelium was intact. Isolated rat aortae showing acetylcholine-induced relaxation from phenylephrine-induced contraction were washed with fresh Krebs solution to restore baseline resting tension. Then, contraction was elicited with isotonic 60 mM KCl as a reference value to compare the magnitude of contraction induced by cumulative addition of phenylephrine in endothelium-intact and endothelium-denuded rat aortae. After contraction was elicited with isotonic 60 mM KCl, the isolated rat thoracic aortae were washed with fresh Krebs solution to restore baseline resting tension prior to the following experimental protocols.

### Experimental protocols

First, we examined the effect of mild hypothermia (33°C) on phenylephrine-induced contraction in endothelium-intact and endothelium-denuded rat aortae. After the isolated endothelium-intact and endothelium-denuded rat aortae were pretreated at 38 or 33°C for 30 min, phenylephrine (10^-9^ to 10^-5^ M) was added cumulatively to the organ bath to produce phenylephrine concentration-response curves. As mild hypothermia (32 to 35°C) is used to reduce neurologic deficits in patients with ruptured intracranial aneurysms, we chose a temperature of 33°C to for the mild hypothermia condition in our study 17.

Second, we examined the effects of the PI3K inhibitor wortmannin (10^-7^ M) and the NO synthase inhibitor L-NAME (10^-4^ M) on phenylephrine-induced contraction in endothelium-intact rat aortae pretreated at 33 or 38°C. After isolated endothelium-intact rat aortae were treated at 38 or 33°C for 30 min in the presence or absence of L-NAME or wortmannin, phenylephrine (10^-9^ to 10^-5^ M) was added cumulatively to the organ bath to produce phenylephrine concentration-response curves.

Third, we examined the effects of wortmannin (10^-7^ M) alone and the combination of L-NAME (10^-4^ M) and wortmannin (10^-7^ M) on the maximal contraction induced by phenylephrine (10^-5^ M) in endothelium-intact rat aortae pretreated at 38 or 33°C for 30 min. After phenylephrine (10^-5^ M) produced maximal sustained and stable contraction in endothelium-intact rat aortae pretreated at 38 or 33°C for 30 min, wortmannin (10^-7^ M) alone or L-NAME (10^-4^ M) followed by wortmannin (10^-7^ M) was added to the organ bath to investigate their effect on the maximal contraction induced by phenylephrine (10^-5^ M) at 38 or 33°C. After the addition of L-NAME (10^-4^ M) produced a sustained and stable effect on maximal phenylephrine (10^-5^ M)-induced contraction, wortmannin (10^-7^ M) was added to the organ bath.

Fourth, we examined the effect of either combined Y-27632 (10^-6^ M), wortmannin (10^-7^ M) and L-NAME (10^-4^ M) or combined L-NAME (10^-4^ M) and Y-27632 (10^-6^ M) on the maximal phenylephrine (10^-5^ M)-induced contraction in endothelium-intact rat aortae pretreated at 38 or 33°C for 30 min. After phenylephrine (10^-5^ M) produced maximal sustained contraction in endothelium-intact rat aortae pretreated at 38 or 33°C for 30 min, either Y-27632 (10^-6^ M), wortmannin (10^-7^ M) and L-NAME (10^-4^ M) or L-NAME (10^-4^ M) and Y-27632 (10^-6^ M) were added sequentially to the organ bath to investigate the cumulative effect of either Y-27632, wortmannin and L-NAME or L-NAME and Y-27632 on the maximal phenylephrine (10^-5^ M)-induced contraction at 38 or 33°C. After the previously added inhibitor produced a sustained and stable effect on maximal phenylephrine (10^-5^ M)-induced contraction, different inhibitors were added to the organ bath.

Finally, we examined the effect of the guanylate cyclase (GC) inhibitor methylene blue (3 × 10^-6^ M) and the NO-sensitive GC inhibitor 1H-[1,2,4]oxadiazolo[4,3-a]quinoxalin-1-one (ODQ, 10^-5^ M) on phenylephrine-induced contraction in endothelium-intact rat aortae pretreated at 33°C for 30 min. After isolated endothelium-intact rat aortae were treated at 33°C for 30 min in the presence or absence of methylene blue or ODQ, phenylephrine (10^-9^ to 10^-5^ M) was cumulatively added to the organ bath to produce phenylephrine concentration-response curves. All the concentrations of various inhibitors, including L-NAME, wortmannin, Y-27632, ODQ and methylene blue, were selected on the basis of previous reports 18,19.

### Cell culture

Human umbilical vein endothelial cells (HUVECs, EA.hy926 cells, American Type Culture Collection, Manassas, VA, USA) were cultured in Dulbecco's modified Eagle's medium (HyClone, GE Healthcare, UT, USA) supplemented with 10% heat-inactivated fetal bovine serum (Gibco, Life Technologies, NY, USA), 2 mM l-glutamine, 100 U/ml penicillin, and 100 µg/ml streptomycin as previously described 20,21. Cells were plated in a 100-mm culture dish and incubated at 37°C in a humidified atmosphere containing 5% CO_2_. The medium was changed every other day until the cells reached confluence. Upon reaching confluence, the cells were dissociated with 0.025% trypsin-ethylenediaminetetraacetic acid solution and split at a 1:4 ratio. For our experiments, cells between passages 2 and 5 were seeded in dishes (10^5^ cells/100-mm dish) and cultured until they reached 70% confluence, followed by serum starvation overnight prior to drug treatment.

### Western blot analysis

Western blot analysis was carried out as described previously 18,19,21. Cytosolic and membrane fractions were isolated from cells using a Mem-PER^®^ eukaryotic membrane protein extraction reagent kit (ThermoFisher Scientific, MA, USA) according to the manufacturer's instructions. Cells were lysed in the appropriate protein extraction solutions to obtain separate cytosolic and membrane fractions by centrifugation at 16,000×*g* for 15 min at 4°C. After extraction, the protein concentrations were determined using the Bradford method. The protein samples to be loaded in the gel were prepared by mixing equal volumes of protein lysates with 2× sodium dodecyl sulfate sample buffer (0.1 M Tris-HCl, 20% glycerol, 4% sodium dodecyl sulfate, and 0.01% bromophenol blue). A total of 30 µg protein per sample was separated by 7% or 10% sodium dodecyl sulfate-polyacrylamide gel electrophoresis for 90 min at 110 V. The separated proteins were electrophoretically transferred to polyvinylidene difluoride membranes for 1 h at 190 mA. Then, the membranes were blocked in Tris-buffered saline containing TWEEN 20 (TBST) with 5% w/v nonfat dried milk for 2 h at room temperature and incubated overnight at 4°C with specific primary antibodies (anti-endothelial nitric oxide synthase [eNOS] and anti-phospho-eNOS) diluted 1:1,000 in TBST containing 5% w/v skim milk powder or 5% BSA. After washing the membranes in TBST, bound antibodies were incubated with horseradish peroxidase-conjugated anti-rabbit or anti-mouse IgG diluted 1:5,000 in TBST containing 5% w/v skim milk for 1 h at room temperature. The membranes were washed in TBST, and the immunoreactive bands were detected by chemiluminescence (SuperSignal^®^ West Pico Chemiluminescent Substrate; Thermo Scientific, Rockford, IL, USA) using X-ray film (*^Super^RX-N* Fuji Medical X-ray Film, Japan).

### Materials

All the chemicals were of the highest purity and obtained from commercial sources. Phenylephrine, L-NAME, wortmannin, ODQ and methylene blue were purchased from Sigma-Aldrich (St. Louis, MO, USA). Y-27632 was obtained from Calbiochem (La Jolla, CA, USA). Anti-eNOS and anti-phospho-eNOS (Ser^1177^) antibodies were obtained from Cell Signaling Technology (Beverly, MA, USA). PRO-PREP protein extraction solution and electrochemiluminescence Western blotting detection reagents were supplied by iNtRON Biotechnology (Houston, TX, USA). All chemical concentrations are expressed as the final molar concentration in the organ bath. The wortmannin and ODQ were dissolved in dimethyl sulfoxide (final organ bath concentration: 0.01%).

### Data analysis

The data are shown as the mean ± SD and are expressed as the percentage of maximal contraction induced by isotonic 60 mM KCl or phenylephrine (10^-5^ M). The logarithm (log ED_50_) of the phenylephrine concentration that induced half of the maximal concentration induced by isotonic 60 mM KCl was calculated using nonlinear regression analysis by fitting the phenylephrine concentration-response curve to a sigmoidal dose-response curve generated by Prism 5.0 (GraphPad Software, San Diego, USA). The effects of mild hypothermia and various inhibitors, alone or combined, on the log ED_50_ and maximal phenylephrine-induced contraction were analyzed using one-way analysis of variance (ANOVA) followed by Bonferroni's post hoc test or an unpaired Student's t-test. The effect of the combined addition of various inhibitors on the phenylephrine-induced maximal contraction at 33 and 38°C was analyzed by repeated-measures ANOVA followed by Bonferroni's post hoc test. The effect of mild hypothermia on the inhibitor-induced change in phenylephrine-induced maximal contraction was analyzed using two-way repeated-measures ANOVA followed by Bonferroni's post hoc test. The effects of mild hypothermia and various inhibitors, alone or combined, on phenylephrine-induced eNOS phosphorylation and endothelial Rho-kinase membrane translocation were analyzed using one-way ANOVA followed by Bonferroni's post hoc test. A *P* value less than 0.05 was considered statistically significant.

## Results

**Figure 1:**
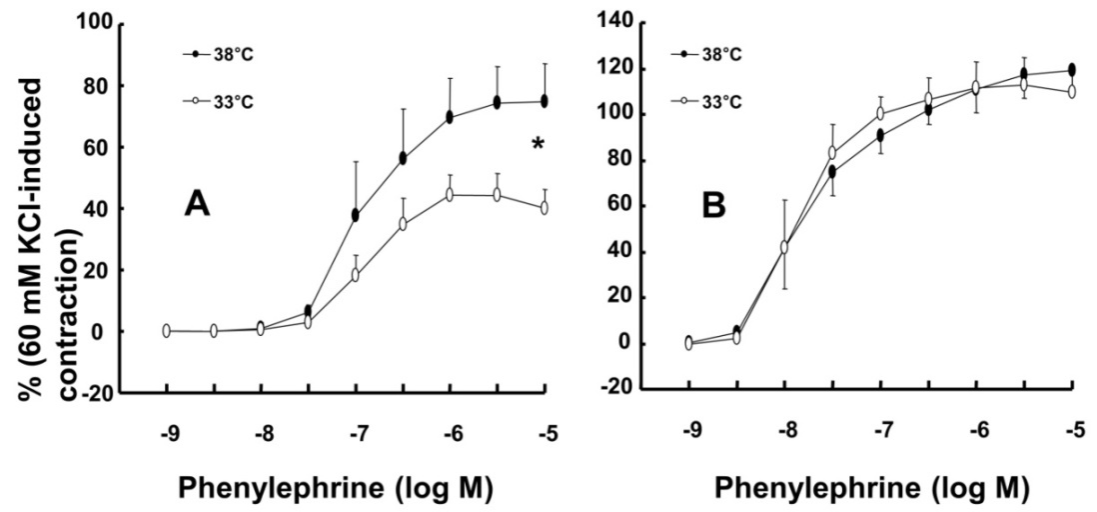
Effect of mild hypothermia (33°C) on phenylephrine-induced contraction in isolated endothelium-intact (A, N = 6) and endothelium-denuded (B, 38°C: N = 7; 33°C: N = 8) rat aortae. The data represent the mean ± SD and are expressed as a percentage of the maximal contraction induced by isotonic 60 mM KCl. N indicates the number of rats (A) or the number of isolated descending thoracic aortic rings (B). Maximal contraction: *P < 0.001 versus 38°C.

**Figure 2:**
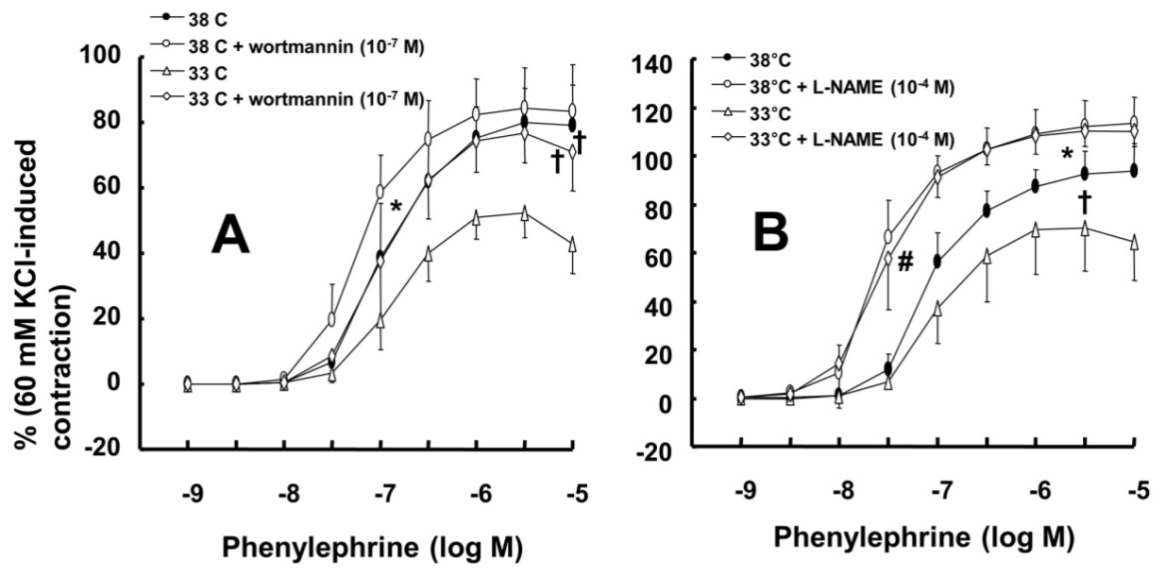
A: Effects of mild hypothermia (33°C) and wortmannin, alone and combined, on phenylephrine-induced contraction in isolated endothelium-intact aortae. The data (N = 6) represent the mean ± SD and are expressed as a percentage of the maximal contraction induced by isotonic 60 mM KCl. N indicates the number of rats from which descending thoracic aortic rings were obtained. Log ED_50_: *P < 0.05 versus 38°C pretreatment only. Maximal contraction: †P < 0.01 versus 33°C pretreatment only. B: Effect of mild hypothermia and N^ω^-nitro-l-arginine methyl ester (L-NAME), alone and combined, on phenylephrine-induced contraction in isolated endothelium-intact aortae. Data (38 or 33°C: N = 5; 38 or 33°C + L-NAME: N = 6) are shown as the mean ± SD and expressed as the percentage of the maximal contraction induced by isotonic 60 mM KCl. N indicates the number of rats from which descending thoracic aortic rings were obtained. Maximal contraction: *P < 0.001 and †*P* < 0.05 versus 33°C pretreatment only. log ED_50_: #*P* < 0.001 versus 38°C or 33°C pretreatment only.

Mild hypothermia (33°C) attenuated phenylephrine-induced maximal contraction in endothelium-intact rat aortae (Fig. 1A; *P* < 0.001 versus 38°C; Table 1) but did not significantly alter phenylephrine-induced contraction in endothelium-denuded rat aortae (Fig. 1B; Table 1). The PI3K inhibitor wortmannin (10^-7^ M) enhanced phenylephrine-induced maximal contraction in endothelium-intact rat aortae pretreated at 33°C (Fig. 2A; *P* < 0.01 versus 33°C pretreatment only; Table 2). In addition, wortmannin caused a leftward shift in the phenylephrine concentration-response curve (Fig. 2A, log ED_50_: *P* < 0.05 versus 38°C alone; Table 2) in endothelium-intact rat aortae pretreated at 38°C but it did not significantly alter phenylephrine-induced maximal contraction (Fig. 2A; Table 2). Pretreatment with the NO synthase inhibitor L-NAME (10^-4^ M) enhanced phenylephrine-induced contraction in endothelium-intact rat aortae pretreated at 38°C or 33°C (Fig. 2B; maximal contraction: *P* < 0.001 versus 33°C pretreatment only; log ED_50_: *P* < 0.001 versus 38°C or 33°C pretreatment only; Table 2). However, when endothelium-intact rat aortae were treated with L-NAME (10^-4^ M), compared with exposure to 38°C, mild hypothermia (33°C) did not significantly alter phenylephrine-induced contraction (Fig. 2B; Table 2).

**Figure 3:**
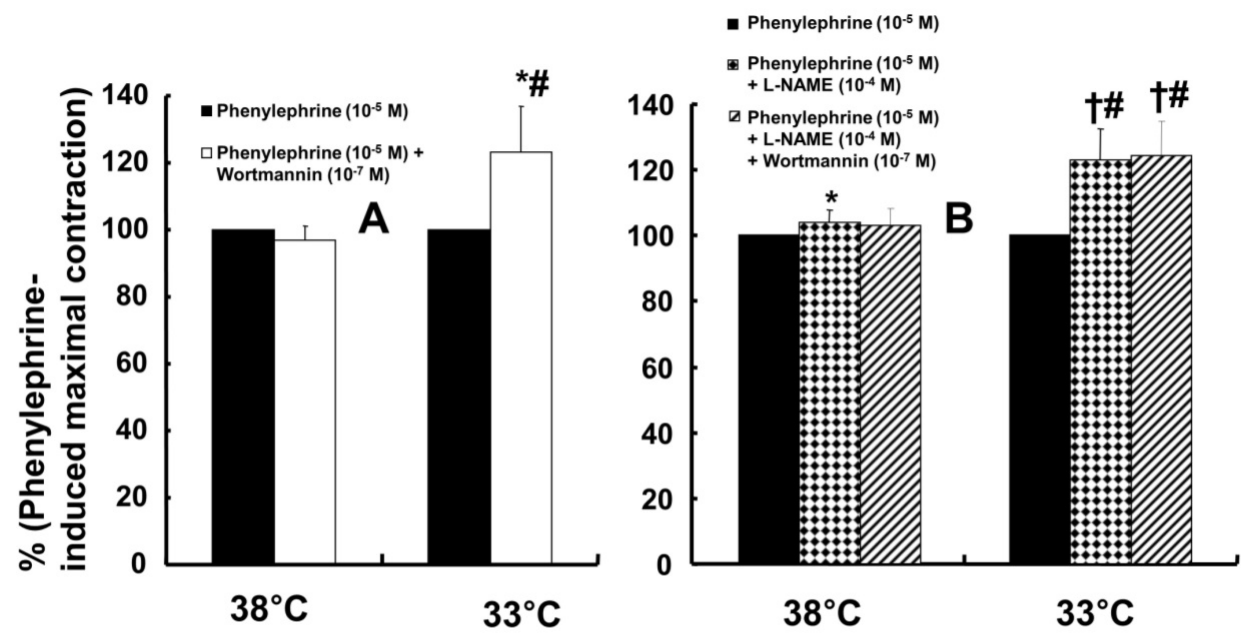
Effects of either wortmannin (**A**; 38°C: N = 6; 33°C: N = 7) or the combination (**B**; 38°C: N = 8; 33°C: N = 10) of N^ω^-nitro-L-arginine-methyl ester (L-NAME, 10^-4^ M) and wortmannin on phenylephrine-induced maximal contraction in isolated endothelium-intact rat aortae pretreated at 33°C or 38°C. The data are shown as the mean ± SD and the percentage of maximal contraction induced by phenylephrine (10^-5^ M). N indicates the number of isolated rat aortae. *P < 0.01 and †*P* < 0.001 versus phenylephrine alone. #*P* < 0.001 versus 38°C.

**Figure 4:**
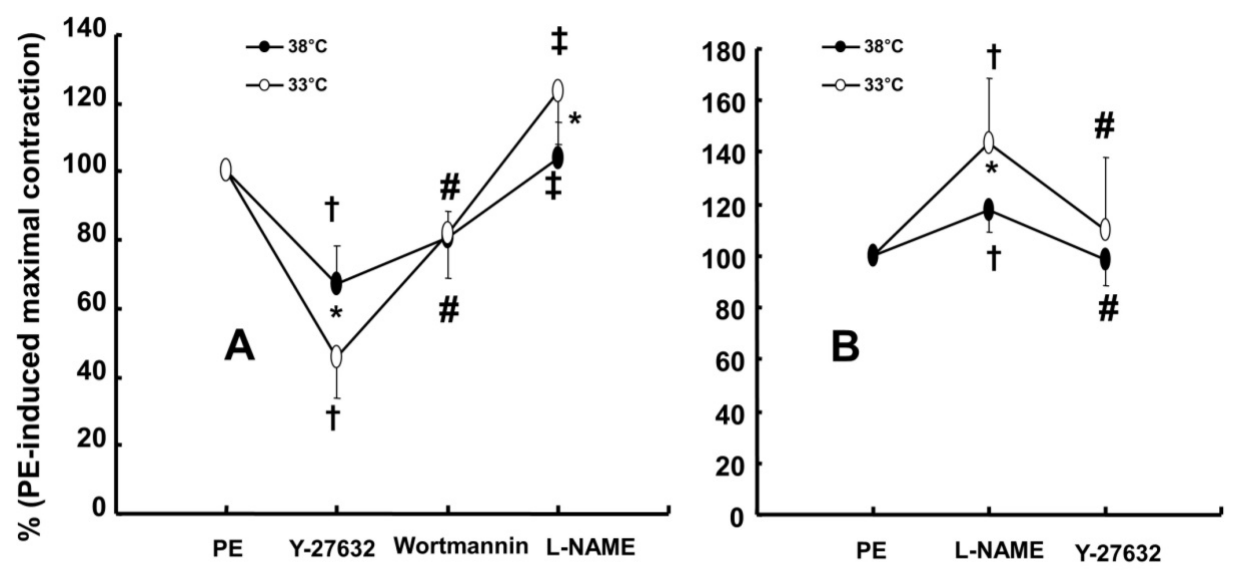
** A:** Effect of cumulative addition of Y-27632 (10^-6^ M), wortmannin (10^-7^ M) and N^ω^-nitro-L-arginine-methyl ester (L-NAME, 10^-4^ M) on maximal contraction induced by phenylephrine (PE, 10^-5^ M) in isolated endothelium-intact rat aortae pretreated at 33°C or 38°C. The data (N = 13) are shown as the mean ± SD and the percentage of maximal contraction induced by PE (10^-5^ M). N indicates the number of isolated rat aortae. *P < 0.001 versus 38°C. †*P* < 0.001 versus PE alone. #*P* < 0.001 versus Y-27632. ‡*P* < 0.001 versus wortmannin. **B:** Effect of combined L-NAME (10^-4^ M) and Y-27632 (10^-6^ M) on maximal contraction induced by PE (10^-5^ M) in isolated endothelium-intact rat aortae pretreated at 33°C (N = 17) or 38°C (N = 18). The data are shown as the mean ± SD and the percentage of maximal contraction induced by PE (10^-5^ M). N indicates the number of isolated rat aortae. *P < 0.001 versus 38°C. †*P* < 0.001 versus PE alone. #*P* < 0.001 versus L-NAME.

**Figure 5:**
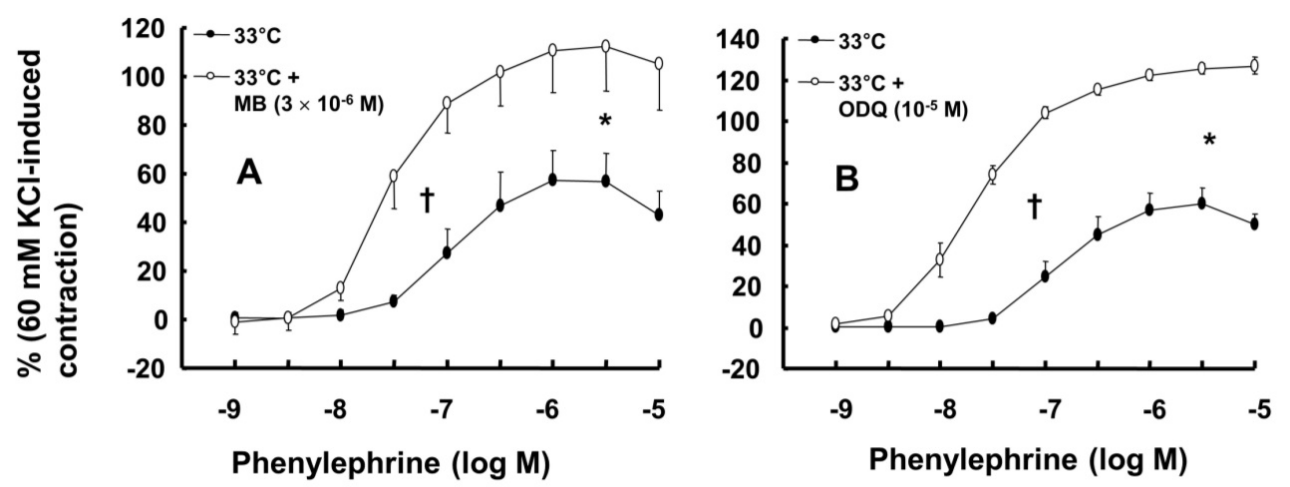
** A:** Effect of methylene blue (MB, A: N = 7) and 1H-[1,2,4]oxadiazolo[4,3-a]quinoxalin-1-one (ODQ, **B:** N = 8) on phenylephrine-induced contraction in isolated endothelium-intact rat aortae pretreated at 33°C. The data represent the mean ± SD and are expressed as a percentage of the maximal contraction induced by isotonic 60 mM KCl. N indicates the number of isolated rat thoracic aortic rings. Maximal contraction: *P < 0.001 versus 33°C alone. Log ED_50_: †*P* < 0.001 versus 33°C alone.

Wortmannin (10^-7^ M) did not significantly alter phenylephrine (10^-5^ M)-induced maximal contraction in endothelium-intact rat aortae pretreated at 38°C (Fig. 3A), but it enhanced phenylephrine (10^-5^ M)-induced maximal contraction in endothelium-intact rat aortae pretreated at 33°C (Fig. 3A; *P* < 0.001 versus phenylephrine alone at 33°C). Posttreatment with L-NAME slightly increased phenylephrine-induced maximal contraction in endothelium-intact rat aortae pretreated at 38°C (Fig. 3B; *P* < 0.001 versus phenylephrine alone at 38°C). Subsequent posttreatment with wortmannin (10^-7^ M) did not significantly alter phenylephrine-induced maximal contraction compared with phenylephrine alone or phenylephrine plus L-NAME. In contrast, posttreatment with L-NAME (10^-4^ M) or L-NAME (10^-4^ M) plus wortmannin (10^-7^ M) remarkably increased phenylephrine-induced maximal contraction in endothelium-intact rat aortae at 33°C (Fig. 3B; *P* < 0.001 versus phenylephrine alone at 33°C); the subsequent addition of wortmannin did not significantly alter the increased phenylephrine-induced maximal contraction induced by L-NAME in endothelium-intact rat aortae at 33°C (Fig. 3B). The increase in phenylephrine-induced maximal contraction induced by L-NAME alone or L-NAME plus wortmannin was more pronounced under hypothermic conditions (33°C) than at 38°C (Fig. 3B; *P* < 0.001). Posttreatment with Y-27632 (10^-6^ M) decreased phenylephrine-induced maximal contraction in endothelium-intact rat aortae (Fig. 4A; *P* < 0.001 versus phenylephrine alone), and subsequent posttreatment with wortmannin (10^-7^ M) increased phenylephrine-induced maximal contraction in endothelium-intact rat aortae treated at 38°C or 33°C (Fig. 4A; *P* < 0.001 versus Y-27632). At both 38°C and 33°C subsequent cumulative posttreatment with L-NAME further increased phenylephrine (10^-5^ M)-induced maximal contraction in endothelium-intact rat aortae that had been previously treated with Y-27632 and wortmannin (Fig. 4A; *P* < 0.001 versus Y-27632 and wortmannin). Hypothermia (33°C) enhanced Y-27632-induced inhibition of phenylephrine (10^-5^ M)-induced maximal contraction in the endothelium-intact rat aortae compared with treatment at 38°C (Fig. 4A; *P* < 0.001). However, after wortmannin-mediated reversal of the effect of Y-27632, there was no longer a significant difference in maximal phenylephrine-induced contraction between aortae treated at 33°C and those treated at 38°C (Fig. 4A). Furthermore, hypothermia (33°C) augmented the ability of L-NAME to increase phenylephrine-induced maximal contraction in endothelium-intact rat aortae pretreated with both Y-27632 and wortmannin (Fig. 4A; *P* < 0.001). Posttreatment with L-NAME (10^-4^ M) increased phenylephrine-induced maximal contraction in endothelium-intact rat aortae pretreated at 38°C or 33°C (Fig. 4B; *P* < 0.001), and subsequent posttreatment with Y-27632 decreased phenylephrine-induced contraction (Fig. 4B; *P* < 0.001 versus L-NAME only). Compared with treatment at 38°C, hypothermia (33°C) augmented the ability of L-NAME to increase phenylephrine-induced maximal contraction in endothelium-intact rat aortae (Fig. 4B; *P* < 0.001), but when Y-27632 was then added, there was no longer a significant difference in phenylephrine (10^-5^ M)-induced maximal contraction in endothelium-intact rat aortae treated at 33°C or 38°C (Fig. 4B). The GC inhibitor methylene blue (3 **×** 10^-6^ M) increased phenylephrine-induced contraction in endothelium-intact rat aortae pretreated at 33°C (Fig. 5A; maximal contraction [33°C + methylene blue: 112 ± 18% versus 33°C: 57 ± 11%] and log ED_50_ [33°C + methylene blue: 7.55 ± 0.10 versus 33°C: 7.00 ± 0.14]: *P* < 0.001 versus 33°C alone). In addition, the NO-sensitive GC inhibitor ODQ (10^-5^ M) increased phenylephrine-induced contraction in endothelium-intact rat aortae at 33°C (Fig. 5B: maximal contraction [33°C + ODQ: 127 ± 4% versus 33°C: 60 ± 8%] and log ED_50_ [33°C + ODQ: 7.65 ± 0.10 versus 33°C: 6.90 ± 0.12]: *P* < 0.001 versus 33°C alone).

**Figure 6:**
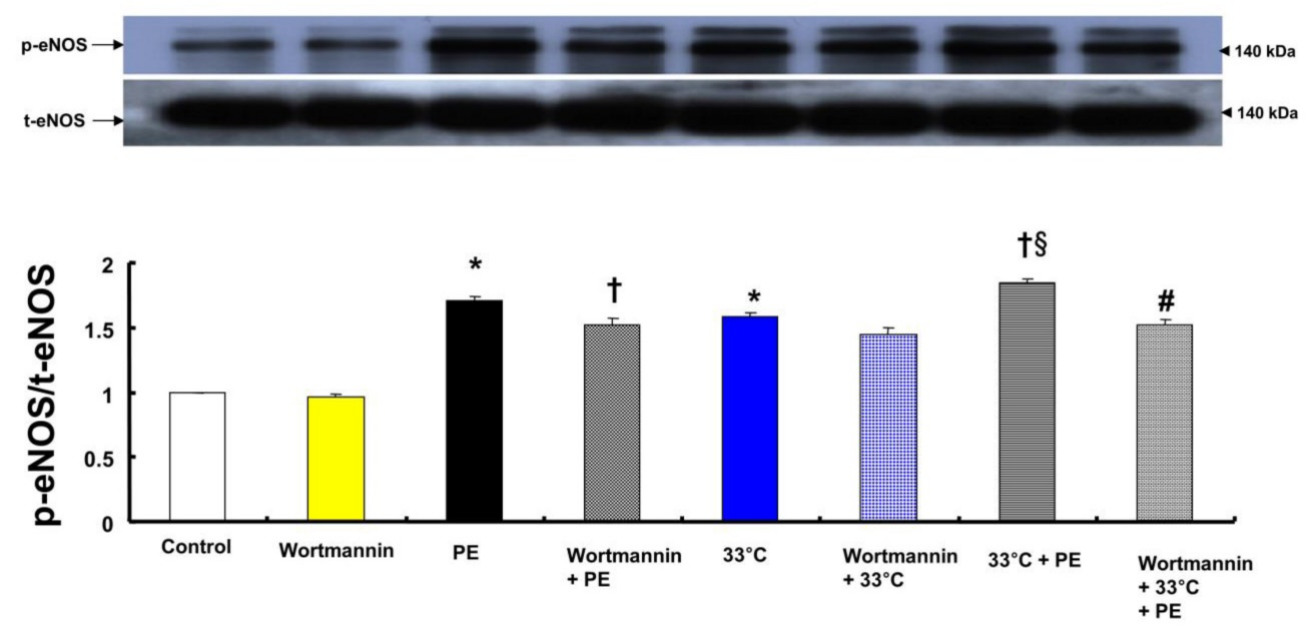
A: Effects of wortmannin and hypothermia, alone and combined, on phenylephrine (PE, 10^-8^ M)-induced endothelial nitric oxide synthase (eNOS) Ser^1177^ phosphorylation in human umbilical vein endothelial cells (HUVECs). HUVECs were treated with wortmannin (10^-7^ M) alone at 37°C for 1 h, PE (10^-8^ M) for 1 min, wortmannin (10^-7^ M) for 1 h followed by PE (10^-8^ M) for 1 min at 37°C, hypothermia (33°C) alone for 41 min, wortmannin (10^-7^ M) at 37°C for 1 h followed by hypothermia (33°C) for 41 min, hypothermia (33°C) for 40 min followed by PE (10^-8^ M) at 33°C for 1 min, or wortmannin (10^-7^ M) at 37°C for 1 h followed by hypothermia (33°C) for 40 min and PE (10^-8^ M) at 33°C for 1 min. Data (N = 3) are shown as the mean ± SD. N indicates the number of experiments. *P < 0.001 versus control. †*P* < 0.01 versus PE alone. §*P* < 0.001 versus 33°C alone. #*P* < 0.001 versus 33°C + PE. p-eNOS: phosphorylated eNOS; t-eNOS: total eNOS.

Both phenylephrine (10^-8^ M) and hypothermia (33°C) induced endothelial nitric oxide synthase (eNOS) Ser^1177^ phosphorylation in HUVECs (Fig. 6; *P* < 0.001 versus control). Moreover, compared with 33°C or phenylephrine alone, combined treatment with 33°C and phenylephrine (10^-8^ M) further increased eNOS (Ser^1177^) phosphorylation (Fig. 6; *P* < 0.001). Pretreatment with wortmannin (10^-7^ M) attenuated the increased phosphorylation of eNOS (Ser^1177^) induced by combined treatment with 33°C and phenylephrine or phenylephrine alone in HUVECs (Fig. 6; *P* < 0.001). L-NAME increased phenylephrine-induced eNOS Ser^1177^ phosphorylation (Supplementary Fig. 1).

**Figure 7:**
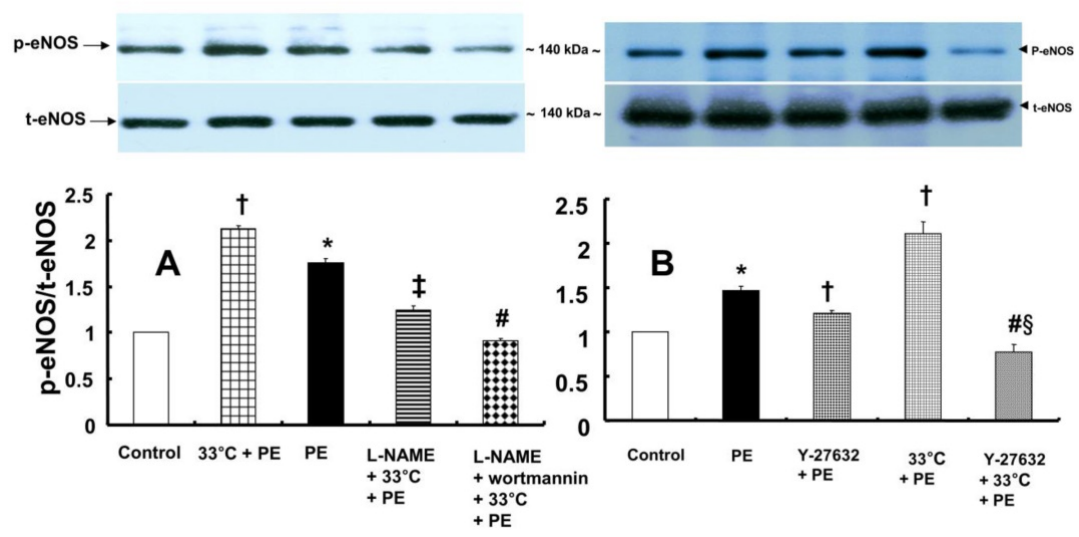
** A:** Effects of hypothermia, N^ω^-nitro-L-arginine-methyl ester (L-NAME, 10^-4^ M) and wortmannin (10^-7^ M), alone and combined, on phenylephrine (PE, 10^-8^ M)-induced endothelial nitric oxide synthase (eNOS) Ser^1177^ phosphorylation in human umbilical vein endothelial cells (HUVECs). HUVECs were treated with PE (10^-8^ M) for 1 min, 33°C exposure for 40 min followed by PE (10^-8^ M) for 1 min at 33°C, L-NAME (10^-4^ M) for 1 h at 37°C followed by 33°C exposure for 40 min and PE (10^-8^ M) for 1 min at 33°C, or L-NAME (10^-4^ M) for 30 min at 37°C followed by 10^-7^ M wortmannin for 30 min at 37°C, 33°C exposure for 40 min and 10^-8^ M PE for 1 min at 33°C. The data (N = 3) are shown as the mean ± SD. N indicates the number of independent experiments. *P < 0.001 versus control. †*P* < 0.001 versus PE alone. ‡*P* < 0.001 versus 33°C + PE. #*P* < 0.001 versus L-NAME + 33°C + PE. p-eNOS: phosphorylated eNOS; t-eNOS: total eNOS. **B:** Effects of Y-27632 and hypothermia, alone and combined, on PE (10^-8^ M)-induced eNOS Ser^1177^ phosphorylation in HUVECs. HUVECs were treated with PE (10^-8^ M) for 1 min, Y-27632 (10^-6^ M) for 1 h followed by PE (10^-8^ M) for 1 min at 37°C, 33°C exposure for 40 min followed by 10^-8^ M PE for 1 min at 33°C, or hypothermia (33°C) for 25 min followed by Y-27632 (10^-6^ M) at 33°C for 15 min and PE (10^-8^ M) for 1 min. The data (N = 3) are shown as the mean ± SD. N indicates the number of independent experiments. *P < 0.001 versus control. †*P* < 0.001 versus PE alone. #*P* < 0.001 versus 33°C + PE. §*P* < 0.001 versus Y-27632 + PE.

**Figure 8:**
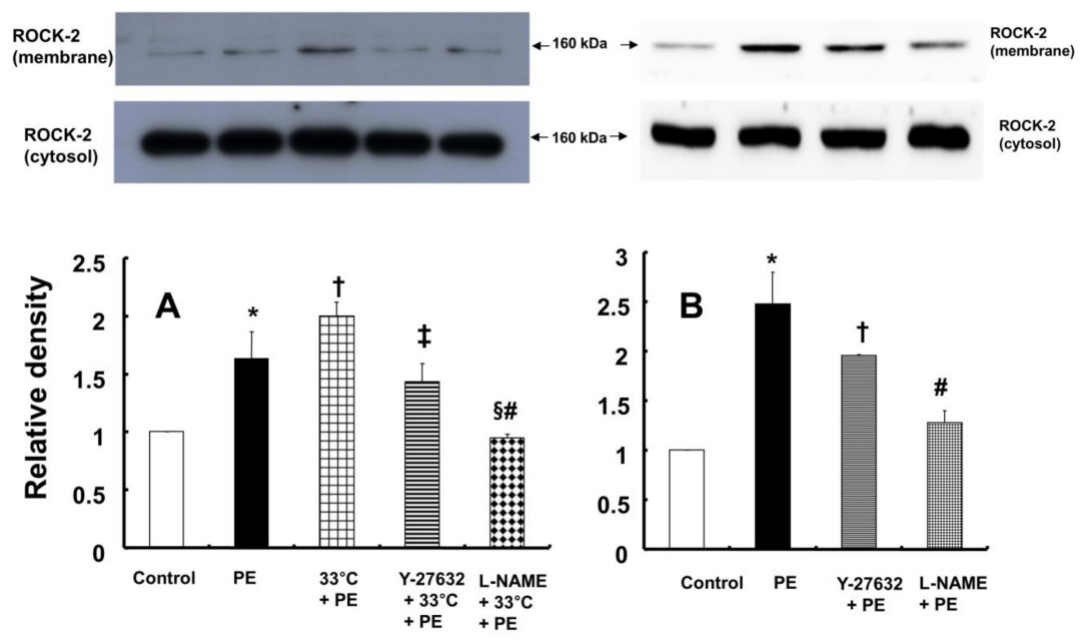
** A:** Effects of hypothermia, Y-27632 (10^-6^ M) and N^ω^-nitro-L-arginine-methyl ester (L-NAME, 10^-4^ M), alone and combined, on phenylephrine (PE, 10^-8^ M)-induced endothelial Rho-kinase (ROCK-2) membrane translocation in human umbilical vein endothelial cells (HUVECs). HUVECs were treated with PE (10^-8^ M) alone for 5 min, pretreated with 33°C exposure for 40 min followed by PE (10^-8^ M) for 5 min at 33°C, or pretreated with either 10^-6^ M Y-27632 or 10^-4^ M L-NAME for 1 h at 37°C followed by 33°C exposure for 40 min and subsequent treatment with 10^-8^ M PE for 5 min at 33°C. The data (N = 3) are shown as the mean ± SD. N indicates the number of independent experiments. *P < 0.01 versus control. †*P* < 0.05 versus PE alone. ‡*P* < 0.01 and §*P* < 0.001 versus 33°C + PE. #*P* < 0.01 versus Y-27632 + 33°C + PE. **B:** Effects of Y-27632 (10^-6^ M) and L-NAME (10^-4^ M) on PE (10^-8^ M)-induced endothelial ROCK-2 membrane translocation at 37°C in HUVECs. HUVECs were treated with PE (10^-8^ M) alone for 5 min or pretreated with either 10^-6^ M Y-27632 or 10^-4^ M L-NAME for 1 h followed by 10^-8^ M PE for 5 min. The data (N = 3) are shown as the mean ± SD. N indicates the number of independent experiments. *P < 0.001 versus control. †*P* < 0.05 and #P < 0.001 versus PE alone.

In contrast, pretreatment with L-NAME (10^-4^ M) attenuated the increased eNOS (Ser^1177^) phosphorylation induced by combined treatment with 33°C and phenylephrine (10^-8^ M) in HUVECs (Fig. 7A; *P* < 0.001). Compared with L-NAME alone, combined treatment with L-NAME and wortmannin further decreased eNOS (Ser^1177^) phosphorylation in aortae that had been previously treated with phenylephrine at 33°C (Fig. 7A; *P* < 0.001). Y-27632 (10^-6^ M) attenuated eNOS (Ser^1177^) phosphorylation induced by phenylephrine alone or combined treatment with hypothermia and phenylephrine (Fig. 7B: *P* < 0.001). However, Y-27632-mediated inhibition of phenylephrine-induced eNOS (Ser^1177^) phosphorylation was more pronounced in mild hypothermia than at 37 °C (Fig. 7B; *P* < 0.001). Phenylephrine induced endothelial Rho-kinase (ROCK-2) membrane translocation in HUVECs (Fig. 8A; *P* < 0.01), and mild hypothermia (33°C) increased phenylephrine (10^-8^ M)-induced Rho-kinase (ROCK-2) membrane translocation (Fig. 8A; *P* < 0.05 versus phenylephrine alone). Both Y-27632 (10^-6^ M) and L-NAME (10^-4^ M) attenuated the enhancement of Rho-kinase (ROCK-2) membrane translocation induced by phenylephrine (10^-8^ M) and hypothermia (Fig. 8A; *P* < 0.01 versus 33°C + phenylephrine). Y-27632 and L-NAME also inhibited phenylephrine (10^-8^ M)-induced ROCK-2 membrane translocation at 37°C (Fig. 8B; *P* < 0.05 versus phenylephrine).

## Discussion

**Figure 9:**
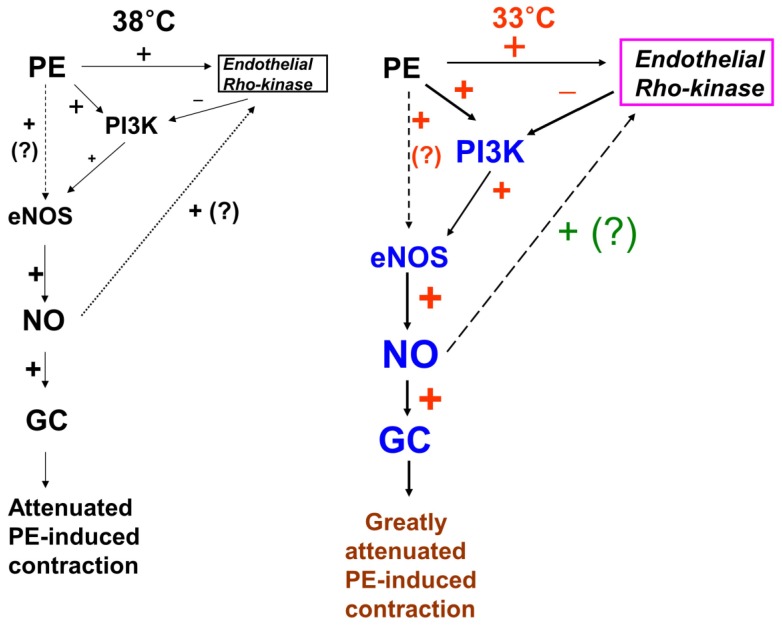
Putative cellular signaling pathways associated with nitric oxide (NO)-mediated inhibition of phenylephrine (PE)-induced contraction in response to mild hypothermia in isolated endothelium-intact rat aortae 6. +: stimulation. -: inhibition. PI3K: phosphoinositide 3-kinase. eNOS: endothelial nitric oxide synthase. GC: guanylate cyclase. ?: unknown pathway. Line thickness and font size of plus or minus signs represent the magnitude of stimulation or inhibition.

This study suggests that inhibition of phenylephrine-induced contraction by hypothermia is mediated by PI3K, which seems to be negatively regulated by endothelial Rho-kinase activation in isolated rat aortae (Fig. 9). The major findings of this study were as follows: 1) mild hypothermia attenuated phenylephrine-induced contraction only in endothelium-intact rat aortae; 2) pretreatment with wortmannin abolished hypothermia-induced inhibition of phenylephrine-induced contraction; 3) posttreatment with wortmannin abolished the enhancing effect of mild hypothermia on the Y-27632-mediated inhibition of phenylephrine-induced contraction (Fig. 4A); 4) wortmannin inhibited hypothermia-induced enhancement of eNOS phosphorylation by phenylephrine in HUVECs; and 5) Y-27632 inhibited hypothermia-induced enhancement of endothelial Rho-kinase (ROCK-2) membrane translocation induced by phenylephrine.

In the current study, the inhibitory effect of mild hypothermia on phenylephrine-induced contraction required an intact endothelium (Fig. 1A and B). L-NAME increased phenylephrine-induced contraction of endothelium-intact aortae at 33°C (Fig. 2B). However, when endothelium-intact aortae were pretreated with L-NAME, there was no significant difference in phenylephrine-induced contraction with mild hypothermia (33°C) compared to 38°C (Table 2). Similar to previous reports, these results suggest that inhibition of phenylephrine-induced contraction in response to mild hypothermia seems to be mediated by enhanced NO production 3,5. Consistent with the results from the current tension study, we found that compared with hypothermia or phenylephrine alone, combined treatment with hypothermia (33°C) and phenylephrine enhanced eNOS phosphorylation (Fig. 6) but that L-NAME inhibited hypothermia (33°C)-induced augmentation of phenylephrine-induced eNOS phosphorylation (Fig. 7A). As hypothermia causes decreased phenylephrine-induced contraction due to increase NO availability, this L-MANE-induced inhibition of eNOS phosphorylation resulting from combined treatment with phenylephrine and hypothermia seems to be associated with a L-NAME-induced decrease in NO production 4,5,22. However, the L-NAME-induced increase in eNOS phosphorylation caused by phenylephrine alone at 37°C may be associated with compensatory eNOS activation due to competitive inhibition of L-NAME against L-arginine as a substrate of NO (supplementary Fig. 1). Furthermore, the GC inhibitor methylene blue and the NO-sensitive GC inhibitor ODQ enhanced phenylephrine-induced contraction in endothelium-intact rat aortae pretreated at 33°C (Fig. 5A and B). Taken together, these results suggest that hypothermia-induced activation of the NO-GC pathway contributes to endothelium-dependent attenuation of phenylephrine-induced contraction.

The PI3K/Akt pathway is involved in endothelial NO production 6,7,22. Similar to previous studies, the current study found that the PI3K inhibitor wortmannin (10^-7^ M) abolished mild hypothermia-induced NO-mediated inhibition of phenylephrine-induced maximal contraction (Fig. 2A) 6,7,23. Furthermore, wortmannin caused the concentration-response curve for phenylephrine-induced contraction to shift to the left at 38°C (Fig. 2A), whereas it greatly increased phenylephrine-induced maximal contraction at 33°C (Fig. 3A). This wortmannin-induced increase in the enhancement of phenylephrine-induced maximal contraction at 33°C seems to be associated with the inhibition of PI3K-induced NO production. However, wortmannin had no effect on L-NAME (10^-4^ M)-induced enhancement of phenylephrine (10^-5^ M)-induced maximal contraction at 33°C (Fig. 3B). In agreement with the results from the tension study, wortmannin inhibited the enhancement of eNOS phosphorylation induced by combined hypothermia and phenylephrine treatment or phenylephrine alone (Fig. 6). Taken together, these results suggest that hypothermia-induced PI3K activity contributes to enhanced NO production, which leads to decreased phenylephrine-induced contraction of endothelium-intact aortae. However, pretreatment with L-NAME followed by treatment with wortmannin in HUVECs attenuated the hypothermia-induced enhancement of phenylephrine-induced eNOS phosphorylation compared with L-NAME alone (Fig. 7A). This inconsistency between the tension study and the Western blot analysis may be due to differences in species (human versus rat) and vessels (aorta versus umbilical artery). Rho-kinase in the vascular smooth muscle induces vasoconstriction by inhibiting MLCP, which leads to increased phosphorylation of the 20-kDa regulatory light chain of myosin 24. Additionally, endothelial Rho-kinase activation decreases endothelial NO release via inhibition of PI3K/Akt 7,11. Thus, hypothermia-induced Rho-kinase activation produces vasoconstriction via both inhibition of MLCP and attenuation of NO release 7,11,13. The Rho-kinase inhibitor Y-27632 potently decreased phenylephrine-induced maximal contraction under mild hypothermia compared with 38°C (Fig. 4A). Cotreatment with Y-27932 and wortmannin has been reported to partially reverse Y-27632-induced inhibition of phenylephrine-induced contraction in endothelium-intact rat aortae but not in endothelium-denuded rat aortae or endothelium-intact aortae pretreated with L-NAME 6. This suggests that Rho-kinase inhibitor-mediated inhibition of phenylephrine-induced contraction is associated with uninhibited PI3K-induced endothelial NO production 6. Thus, considering previous reports, the increased Y-27632-induced inhibition of phenylephrine-induced maximal contraction observed at 33°C (Fig. 4A) may be due mainly to increase NO production via increased activation of PI3K 6. As in a previous report, subsequent treatment with wortmannin abolished the enhancing effect of Y-27632 on the inhibition of maximal phenylephrine-induced contraction observed at 33°C (Fig. 4A) 6. Taken together, these results suggest that, as hypothermia enhanced endothelial Rho-kinase membrane translocation induced by phenylephrine (Fig. 8A), mild hypothermia (33°C)-induced endothelial Rho-kinase membrane translocation contributes to enhanced contraction via PI3K inhibition-mediated decreased NO production 6,7,11,13. This response may be associated with a compensatory mechanism to counterbalance hypothermia-induced endothelial NO production. In endothelium-intact aortae pretreated with Y-27632 and wortmannin, the subsequent addition of L-NAME enhanced phenylephrine-induced contraction under conditions of mild hypothermia compared with 38°C. This result suggests that other NO production pathways that are not mediated by PI3K may contribute to the decrease in phenylephrine-induced maximal contraction in mild hypothermia. L-NAME enhanced phenylephrine-induced maximal contraction in mild hypothermia compared with 38°C, but after addition of the Rho-kinase inhibitor Y-27632, contraction decreased to similar levels at both 33°C and 38°C (Fig. 4B). This lack of significant difference in phenylephrine-induced maximal contraction between 38 and 33°C with the addition of Y-27632 following pretreatment with L-NAME (Fig. 4B) may be due to increased inhibition of mild hypothermia-induced, Rho-kinase-mediated enhancement of vascular smooth muscle cell contraction caused by the relative activation of MLCP in the vascular smooth muscle 13,24. Consistent with the tension study, Y-27632 attenuated the hypothermia-induced enhancement of endothelial Rho-kinase membrane translocation induced by phenylephrine (Fig. 8A). However, pretreatment with L-NAME caused greater inhibition of hypothermia- and phenylephrine-induced enhancement of endothelial Rho-kinase membrane translocation compared with pretreatment with Y-27632 (Fig. 8A). Reciprocally, Y-27632-mediated inhibition of phenylephrine-induced eNOS phosphorylation was enhanced in mild hypothermia conditions compared with 37 °C (Fig. 7B). These results suggest that the putative underlying mechanism for L-NAME's inhibition of endothelial Rho-kinase membrane translocation increased by phenylephrine and hypothermia (Fig 8A) and Y-27632's inhibition of phenylephrine- and hypothermia-induced eNOS phosphorylation (Fig. 7B) is as follows 25. Given that mild hypothermia (33 °C) enhanced phenylephrine-induced eNOS phosphorylation and ROCK-2 membrane transloncation (Fig 6 and 8A) and that the ability of mild hypothermia to attenuate phenylephrine-induced contraction involves PI3K-mediated endothelial NO release (Fig. 3A and B) and Rho-kinase activation (Fig. 4A), enhanced NO release in hypothermia may activate endothelial Rho-kinase to counterbalance excessive NO-mediated attenuation of phenylephrine-induced contraction (Fig. 9) 7,13. Thus, pretreatment with L-NAME at 33°C may contribute to decreased Rho-kinase membrane translocation through the inhibition of NO production induced by mild hypothermia. On the other hand, activation of the NO-cGMP pathway inhibits the RhoA/ROCK pathway 25. The relationship between the Rho-kinase pathway and the NO-cGMP pathway in the mildly hypothermic endothelium remains to be characterized in detail.

The clinical relevance of this study is as follows. When hypotension is encountered during therapeutic mild hypothermia, drugs that inhibit the NO-GC-cGMP pathway, including methylene blue, may be effective in restoring normal blood pressure. In addition, as statins, which lower cholesterol levels, attenuate the activity of Rho-kinase, patients concurrently taking statins during therapeutic mild hypothermia may face more severe hypotension than patients not taking statins 11. The limitations of this study are as follows. First, small resistance arterioles are the main determinants of vascular resistance and contribute to blood pressure, but the aorta, a large conduit artery, was used in the current study 26. Second, rat aortae were used for the tension study, whereas HUVECs were used to detect eNOS phosphorylation and Rho-kinase membrane transloncation. Third, this *in vitro* study did not consider the heart or the nervous system, which are important for regulating hemodynamics *in vivo*. Fourth, based on the previous reports, 38°C was used for tension experiment to compensate for heat loss during circulation of heated water in the organ bath 3,5. However, 37°C was used for experiment using HUVECs.

In conclusion, mild hypothermia attenuated phenylephrine-induced contraction in isolated rat aortae via the NO-GC pathway, partially through regulation by PI3K, and this phenomenon seems to be inhibited by endothelial Rho-kinase (Fig. 9).

## Supplementary Material

Supplementary figure S1.

## Figures and Tables

**Table 1:** Effect of mild hypothermia on phenylephrine-induced contraction in isolated rat aortae

	Endothelium-intact aortae			Endothelium-denuded aortae		
	N	LogED50	Max contraction (%)	N	LogED50	Max contraction (%)
38°C	6	6.92 ± 0.23	75 ± 12	7	7.83 ± 0.21	119 ± 10
33°C	6	6.90 ± 0.13	44 ± 7*	8	7.86 ± 0.21	110 ± 9

Data are shown as the mean ± SD. N indicates the number of rats for the endothelium-intact condition or the number of isolated aortae for the endothelium-denuded condition. Log ED_50_: Logarithm of phenylephrine concentration to produce half of the maximal contraction induced by isotonic 60 mM KCl. Max contraction: Maximal contraction induced by phenylephrine, which is expressed as the percentage of isotonic 60 mM KCl-induced maximal contraction. *P < 0.001 versus 38*°*C.

**Table 2:** Effect of L-NAME and wortmannin on phenylephrine-induced contraction in isolated endothelium-intact rat aortae pretreated with 38 or 33°C

	38°C			33°C		
	N	LogED_50_	Max contraction (%)	N	LogED_50_	Max contraction (%)
Control	6	6.94 ± 0.16	80 ± 11‡	6	6.89 ± 0.13	53 ± 8
Wortmannin (10-7 M)	6	7.21 ± 0.11*	84 ± 14‡	6	6.99 ± 0.20	76 ± 11†
Control	5	7.04 ± 0.12	94 ± 11§	5	7.00 ± 0.14	70 ± 14
L-NAME (10-4 M)	6	7.58 ± 0.14#	113 ± 11**	6	7.55 ± 0.23#	110 ± 6#

Data are shown as the mean ± SD. N indicates the number of rats. Log ED_50_: Logarithm of phenylephrine concentration to produce half of the maximal contraction induced by isotonic 60 mM KCl. Max contraction: Maximal contraction induced by phenylephrine, which is expressed as the percentage of isotonic 60 mM KCl-induced maximal contraction. *P < 0.05, †*P* < 0.01 and #*P* < 0.001 versus control. §*P* < 0.05, ‡*P* < 0.01 and **P <0.001 versus 33°C pretreatment only.

## Abbreviations

NO: nitric oxide
cGMP: cyclic guanosine monophosphate
PI3K: phosphoinositide 3-kinase
MLCP: myosin light chain phosphatase
HUVEC: human umbilical vein endothelial cell
eNOS: endothelial nitric oxide synthase
GC: guanylate cyclase
L-NAME: N^ω^-nitro-L-arginine methyl ester
ODQ: 1H-[1,2,4]oxadiazolo[4,3-a]quinoxalin-1-one
