# Supplementary material for: Nitric oxide-mediated inhibition of phenylephrine-induced contraction in response to hypothermia is partially modulated by endothelial Rho-kinase
Source: Int J Med Sci. 2020 Jan 1;17(1):21–32. doi: 10.7150/ijms.39074 (PMC6945562; doi:10.7150/ijms.39074)
Supplement: Supplementary file 1 — Supplementary figure S1. [file ijmsv17p0021s1.pdf]

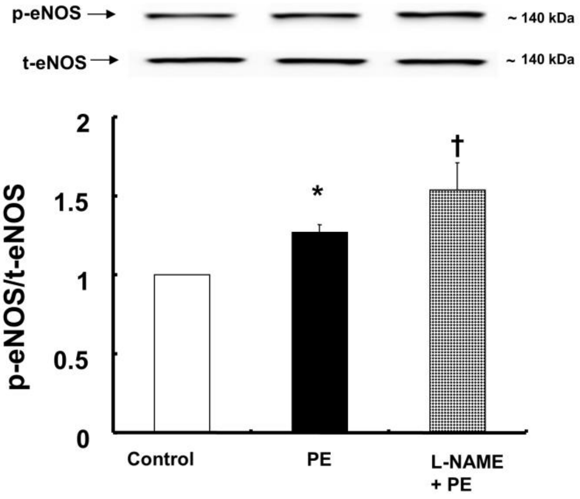

Supplementary Figure 1

Supplementary Figure 1. Effects of N^{ω}-nitro-L-arginine methyl ester

(L-NAME, 10^{-4} M) on phenylephrine (PE, 10^{-8} M)-induced endothelial nitric oxide synthase (eNOS) Ser^{1177} phosphorylation in human umbilical vein endothelial cells (HUVECs) at 37°C. HUVECs were treated with PE (10^{-8} M) for 1 min or L-NAME (10^{-4} M) for 1 h followed by PE (10^{-8} M) for 1 min. Data (N = 4) are shown as the mean ± SD. N indicates the number of experiments. *$P < 0.05$ versus control. †$P < 0.05$ versus PE alone. p-eNOS: phosphorylated eNOS; t-eNOS: total eNOS.
